# Synthesis and Characterization of New Polycarbonate-Based Poly(thiourethane-urethane)s

**DOI:** 10.3390/polym14142933

**Published:** 2022-07-20

**Authors:** Andrzej Puszka, Janusz W. Sikora

**Affiliations:** 1 Department of Polymer Chemistry, Faculty of Chemistry, Institute of Chemical Sciences, Maria Curie-Skłodowska University in Lublin, Gliniana 33, 20-614 Lublin, Poland; 2 Department of Technology and Polymer Processing, Faculty of Mechanical Engineering, Lublin University of Technology, Nadbystrzycka 36, 20-618 Lublin, Poland; janusz.sikora@pollub.pl

**Keywords:** thermoplastic polyurethanes, surface free energy, dithiol, mechanical properties, differential scanning calorimetry, optical properties

## Abstract

The new segmented poly(thiourethane-urethane)s (PTURs) based on 1,1′-methanediylbis(4-isocyanatocyclohexane) (HMDI, *Desmodur W*^®^), polycarbonate diol (PCD, *Desmophen C2200*) and (methanediyldibenzene-4,1-diyl)dimethanethiol were synthesized by one-step melt polyaddition method. The obtained PTURs, with a content of 30–60 wt% of the hard segments (HS), were tested in which the influence of changes in the HS content on their properties was determined. The polymers were characterized by Fourier transform infrared spectroscopy (FTIR), gel permeation chromatography (GPC), thermal analysis (DSC, TGA) and thermomechanical analysis (DMTA). Additionally, tensile strength, optical (refractive index, UV-VIS and color) and surface properties of the obtained polymers (contact angle and surface free energy) and adhesion to copper were examined. FTIR analysis verified the supposed structure of the polymers obtained and showed a complete conversion of the isocyanate groups. TGA analysis confirmed the relatively good thermal stability of the polymers. On the other hand, after performing the DSC analysis, it was possible to state that the obtained materials were partially or completely amorphous, and the microphase separation decreased with increasing HS content in the polymer. Similar observations were made from the DMTA data. In addition, the hardness, tensile strength, modulus of elasticity, storage modulus, adhesion to copper, refractive index and total free surface energy increased with increasing HS content in the polymer.

## 1. Introduction

Polyurethanes (PUs) are an important group of polymers that have a number of unique properties [1,2,3,4], which provides opportunities for their use in various industries, e.g., packaging, electronic, automotive, construction, building and medicine [5,6,7,8,9,10,11]. The various applications of polyurethanes also include the modification of textiles in order to provide them with increased mechanical strength and water resistance [12,13]. Among polyurethane materials, we can distinguish foams, adhesives, varnishes, fibers, elastomers, etc. [5]. The PUs can also form suitable blends [14,15,16,17,18] or composites [19,20,21,22,23].

PUs are generally formed by reacting diisocyanates with polyols or diamines [2]. Flexible polyol macro-chains have low glass transition temperatures (*T_g_*) and form soft segments (SS), while hydrogen-bonded urethane linkages create hard segments (HS) of the polymer network structure [1,2,3,4,7,8,13]. These segments may be self-soluble with good mixing or may remain separate as segmented polyurethane (SPU)-forming domains. The quantities and separations of HS and SS determine the properties of the formed polymer. HS ensures a high modulus, while SS ensures the extensibility of the product [3,4]. Hydrogen bonding among hard and hard segments (HS-HS) and hard and soft segments (HS-SS) is created due to the presence of polar functional N-H and C=O groups on PU chains. The level of miscibility of HS and SS relies on the amount of hydrogen bonding between these segments [2,7,8,13]. In situations where there is a stronger hydrogen bond between HS and HS, phase separation is predominant, resulting in high performance products [1,3]. The phase intermixing is due to strong intra- or intermolecular interactions between HS and SS. Indeed, this is a key element highlighting the relevance of the 3D positioning of the functional groups [24], which has a significant influence on the mechanical properties of the final product. SPUs with different properties can be obtained by varying the type and the amount of initial components, by using different molecular weight diol component or by changing the preparation process. All these mechanisms change the amount and distribution of the HS and SS and lead to variation in the final properties [10,11]. This aspect of molar mass variation for polymeric network has been highlighted as well by De Keer et al. [25].

**Figure 1:**
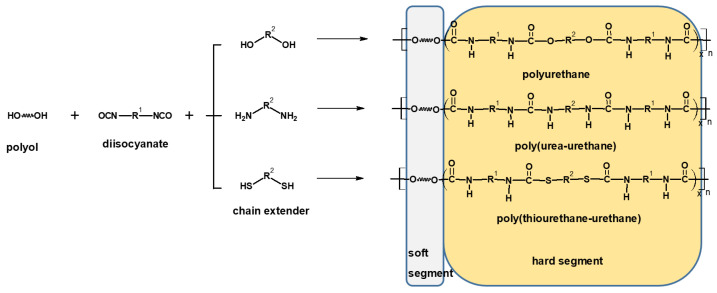
General scheme of reactions between polyol, diisocynate and different chain extenders.

The soft segments consist of polyether, polyester or polycarbonate polyols, while the hard segments are formed by reactions between a diisocyanate and low molecular weight diol, dithiol or diamine chain extender (see Figure 1).

Proper selection of the appropriate flexible segments in the case of biomedical materials is extremely important due to the scope of their potential application. This choice determines the hydrolytic stability of the SS. The issue of the hydrolytic stability of polyurethane materials has been the subject of many authors’ research for many years [26,27,28]. The first PUs used in medicine were synthesized with the use of polyester diols, but the presence of ester bonds made them highly hydrolytic unstable. The chemical structure, and above all a highly developed surface, easily accessible to organic fluids, cells and enzymes, causes rapid hydrolytic degradation of these polymers. The resulting carboxyl groups reduce the pH value locally, additionally catalyzing their further destruction. For this reason, the polyester component in PUs has been replaced by a polyether component which is resistant to hydrolysis [28]. However, research has shown that poly(ether-urethane)s are in turn susceptible to so-called oxidative degradation, which is the result of the joint interaction of body fluids, oxidizing compounds and stresses. A mechanism for this degradation has been proposed, involving the initiation of the process by adhesion and activation of cells (phagocytes), followed by the release of oxidizing substances (HOO·) that attack the polymer in the polyether segment [28]. A mechanism that may additionally occur during the degradation of such PUs under physiological conditions is acid hydrolysis. Such slow hydrolysis can take place, for example, by the action of hydrogen ions secreted by leukocytes. In this reaction, the oxygen atom of the PUs ether group undergoes protonation, which leads to chain breakage. This course of the reaction is consistent with reports that PUs based on polyethers are much more easily degraded in an acidic environment than poly(carbonate-urethane)s. For these reasons, hydrolytically and oxidatively stable oligomeric polycarbonate diols are used as the polyol component of the newest generation of PUs, which in turn made it possible to obtain biologically stable poly(carbonate-urethane)s [28]. The hydrolytic stability of polycarbonates is associated with low water permeability, which is attributed to the rigidity of the polymer chains. Moreover, unlike polyesters, the hydrolysis of the carbonate bond leads to the formation of two groups, -OH and CO_2_, i.e., products that do not further catalyze polycarbonate hydrolysis [26,27,28].

Another fragment of polyurethane materials that can be hydrolyzed is the HS fragment, which is much more resistant to degradation than the soft segment. As it results from the available literature, amine, alcohol and CO_2_ are formed during the hydrolysis of urethane bonds. In turn, urea bonds are hydrolyzed to amines and CO_2_.

For special applications, other chain extenders can be used that contain reactive hydrogen atoms in their structure, e.g., dithiols [29,30,31,32,33,34]. The appearance of sulfur atoms in the polymer structure, depending on the type of functional group, can enhance some key properties, e.g., thermal and chemical resistance and optical properties (refractive index). The use of chain extenders containing sulfur atoms in their structure is the subject of mine and my colleagues’ research. Of the many systems available, we have synthesized derivatives of diphenylmethane [33,34,35,36,37,38,39,40,41,42,43,44,45,46,47], diphenylethane [48,49,50,51,52], benzophenone [53,54,55], diphenyl ether [56] and diphenyl sulphide [38,57,58,59,60]. In our previous studies, we have attempted to obtain and investigate the effects of such unconventional chain extenders on the structural, thermal, mechanical and optical properties of novel polyurethane materials. Polymers obtained with the use of unconventional chain extenders were characterized by hardness up to 81/61 Sh A/D and tensile strength up to 48.9 MPa, and these values are higher than those of the commercial Chronoflex^®^ AL 80A biomaterial. Such materials were also characterized by better optical properties (refractive index 1.525 vs. 1.501, transmittance 88.5% vs. 82.1%) compared to polyurethanes obtained with butane-1,4-diol as the chain extender.

In this study, various poly(thiourethane-urethane)s (PTURs) were synthesized by using cycloaliphatic 1,1′-methanediylbis(4-isocyanatocyclohexane) (*Desmodur W^®^*, HMDI) and sulfur-containing chain extender (methanediyldibenzene-4,1-diyl)dimethanethiol (dithiol), forming hard segments in the obtained polymers. The soft segments were composed of PCD diol (*Desmophen^®^ C2200*). By applying the one-step melt method, we obtained PTURs with HS contents of 30, 40, 50 and 60 wt%. The influence of the composition on the properties of the obtained PTURs was investigated. For the synthesized polymers, using Fourier transform infrared spectroscopy (FTIR), the physicochemical (reduced viscosities, gel permeation chromatography (GPC), contact angles (CAs) and surface free energy (SFE), optical (refractive index, transparency and color), thermal (differential scanning calorimetry (DSC) and thermogravimetric analysis (TGA)), thermomechanical (dynamic thermal mechanical analysis (DMTA)), mechanical (hardness and tensile test) and adhesive properties have been studied.

## 2. Materials and Methods

### 2.1. Materials

Dithiol (T_m_ = 85 °C) was obtained at the Department of Polymer Chemistry, Maria Curie-Skłodowska University [33,61]. HMDI (*Desmodur W*^®^) and PCD of $\bar{M_{n}}$ = 2000 g/mol (*Desmphen^®^ C2200*) were kindly supplied by Covestro (Leverkusen, Germany). Before its use, the PCD was heated at 90 °C in vacuo for 10 h. Dibutyltin dilaurate (DBTDL) from Merck-Schuchardt (Hohenbrunn, Germany) was used as received. The analytical reagents were as follows: 1,1,2,2-tetrachloroethane (TChE), diiodomethane and tetrahydrofuran (THF) (Aldrich, St. Louis, MO, USA), 1–bromonaphthalene (POCh S.A., Gliwice, Poland), redistilled water (Millipore, UMCS, Lublin, Poland) and Optylite^®^ physiological saline (KabiPac, Kutno, Poland) were used in purchased form.

### 2.2. Measurements Methods

#### 2.2.1. Fourier Transform Infrared Spectroscopy (FTIR)

FTIR spectra were obtained using the attenuated total internal reflection (ATR/FTIR) method with a Bruker TENSOR 27 spectrophotometer (Ettlingen, Germany) equipped with a PIKE measuring cell with crystalline diamond embedded in zinc selenide. The FTIR spectra were recorded from 4000 to 600 cm^−1^, at a resolution of 2 cm^−1^ and with 128 scans per sample in the absorption mode. The PTURs were in the form of a 1 mm-thick pressure-formed sheet.

#### 2.2.2. Physicochemical Characterization

1. Gel permeation chromatography (GPC)

The number ($\bar{M_{n}}$) and mass ($\bar{M_{w}}$) average molar mass (Daltons (Da))), and the molar mass dispersity (*Ð*_M_, *Ð*_M_ = $\bar{M_{w}}$/$\bar{M_{n}}$) [62] of the PTURs were determined by gel permeation chromatography (GPC) carried out on a Viscotek GPCMax (Westborough, MA, USA) equipped with Triple Detector Array TDA305. The eluent, with the flow rate 1 mL/min, was tetrahydrofuran (THF), and the operation temperature was set to be 35 °C. The molar mass was calibrated with polystyrene standards.

2. Reduced viscosities

Reduced viscosities (*η*_red_, dL/g) of 0.5% the polymer solution in (TChE) were determined in an Ubbelohde viscometer (Gliwice, Poland) at 25 °C.

3. Contact angles (CAs) and Surface Free Energy (SFE)

Contact angles (CAs) of PTURs were measured at 20 °C with a contact angle goniometer (KRÜSS GmbH, Hamburg, Germany) with water and diiodomethane droplets. The volume of droplets was 2 μL. Each sample was analyzed five times and the average value of the contact angle was designated. For calculation of the surface free energy according to the method of Owens, Wendt, Rabel and Kaelble [63], the Krüss ADVANCE (KRÜSS GmbH, Hamburg, Germany) software was used. The sessile drop orientation and the ellipse fitting method were used for data analysis.

4. Hydrolytic resistance

The PTURs (weighing approximately 1 g) were placed in Optylite^®^ saline. Its ion concentration was as follows (mmol/dm^3^): 141 Na^+^, 34 CH_3_COO^−^, 3 C_6_H_5_O_7_^3−^, 2 Ca^2+^, 5 K^+^, 1 Mg^2+^, 109 Cl^−^. The solution temperature was 37 °C and the immersion time was 8 weeks. After each week, the samples were taken from the solution, washed in distillated water and dried in vacuum at 60 °C. Then, changes in their mass (%) were determined.

#### 2.2.3. Thermal and Thermomechanical Properties

1. Differential Scanning Calorimetry (DSC)

DSC analysis was carried out using a Netzsch 204 F1 Phoenix calorimeter (Günzbung, Germany), in accordance with the standard ISO 11357-1:2016 [64]. Each sample (weighing about 10 mg) was analyzed with the following program: cooling and isotherm for 3 min at −100 °C, heating to 200 °C, next cooling to −100 °C and then heating to 200 °C. The test was carried out at a heating rate of 10 °C/min, and under an argon atmosphere (flow 30 mL/min. Aluminum crucibles with a pierced lid were used as measuring cells, while an analogous empty crucible was used as reference. The glass transition temperatures (*T_g_*) were read from the inflection point of the DSC curves, and the melting temperatures (*T_m_*) from the maximums of the endothermic peaks.

2. Thermogravimetric Analysis (TGA)

TGA was carried out using a Netzsch STA 449 F1 Jupiter thermal analyzer (Selb, Germany) in a helium environment (gas flow = 20 cm^3^/min), with the temperature ranging from 30 to 800 °C and a heating rate of 10 °C/min. Polymer samples weighing about 10 mg were placed in an aluminum(III) oxide (Al_2_O_3_) crucible and an analogous crucible was used as the reference.

3. Dynamic Mechanical Thermal Analysis (DMTA)

DMTA of PTURs was performed in tensile mode using DMA Q800 Analyzer TA Instruments (New Castle, DE, USA). Calibration was performed as per the manufacturer’s recommendations included in Advantage Software, version 5.5.24 (TA Instruments, New Castle, DE, USA). The experiments were carried out on rectangular samples of dimensions close to 1 mm thick, 5 mm wide and 30 mm long. Polymer shapes were prepared using the Carver hydraulic press with heated plates. Press molding was performed at 100–120 °C under a 10–30 MPa pressure. Experimental conditions employed were a frequency of 1 Hz and static stain of 0.05%, with the scanning temperature ranging from −100 °C to 150 °C in the air conditions and a temperature ramp of 3 °C/min. The samples were cut from the pressed sheets. The variations in storage modulus (*E*′), loss modulus (*E*″) and tangent delta (tan*δ*) versus temperature were determined.

#### 2.2.4. Mechanical Properties

Tensile testing was carried out using a Zwick/Roell Z010 tensile-testing machine (Ulm, Germany) agreeing to the International Standard ISO 527-2:2012 [65] at the speed of 100 mm/min at 23 °C. Polymeric samples (1 mm thick and 6 mm wide for the section measured) were cut from the pressed sheet. Polymer shapes were prepared using the Carver hydraulic press with heated plates. Press molding was performed at 100–120 °C under a 10–30 MPa pressure. The hardness of the PTURs was determined by the Shore A/D method using a Zwick 7206/H04 hardness tester (Ulm, Germany). The readings were taken after 15 s at the temperature of 23 °C [66].

The single lap-shear strength of polymers on a copper plate with dimensions of 100 mm × 25 mm × 1.5 mm was measured in accordance with the Polish Standard PN EN 1465: 2009 [67] using the Zwick/Roell Z010 device (Ulm, Germany). An adhesive joint from a polymer sample of 12.5 mm × 25 mm × 0.2 mm dimensions was prepared by pressing the polymer between two copper plates (prepared according to PN-EN-13887:2005 [68]). The strength of such an adhesive joint was measured at the tensile speed of 2 mm/min at 23 °C.

#### 2.2.5. Optical Properties

1. Refractive index (RI)

RI was measured by using Conbest Abbe’s Refractometer Type 325 (Krakow, Poland) instrument according to Method A of the European Standard EN ISO 489:2022 [69] at 23 °C. The contact liquid was 1–bromonaphthalene.

2. Transmittance

The ultraviolet-visible (UV/vis) spectra of the PTURs were obtained by a UV-2550 (Shimadzu, Kioto, Japan) UV spectrophotometer at a scanning rate of 200 nm/min and in the range of 200–900 nm.

3. Color

The color of the polymers was determined using a X-Rite Ci4200 spectrophotometer in accordance with the standard ASTM E308 [70]. The color is defined in the CIELab system, where it is specified in L*, a*, b* space. Parameter a* is the color from green (negative values) to red (positive values); parameter b* is the color from blue (negative values) to yellow (positive values); and parameter L* is the brightness, describing the grey scale from black to white (value 0 corresponds to black and 100 to white).

### 2.3. Polymer Synthesis

**Figure 2:**
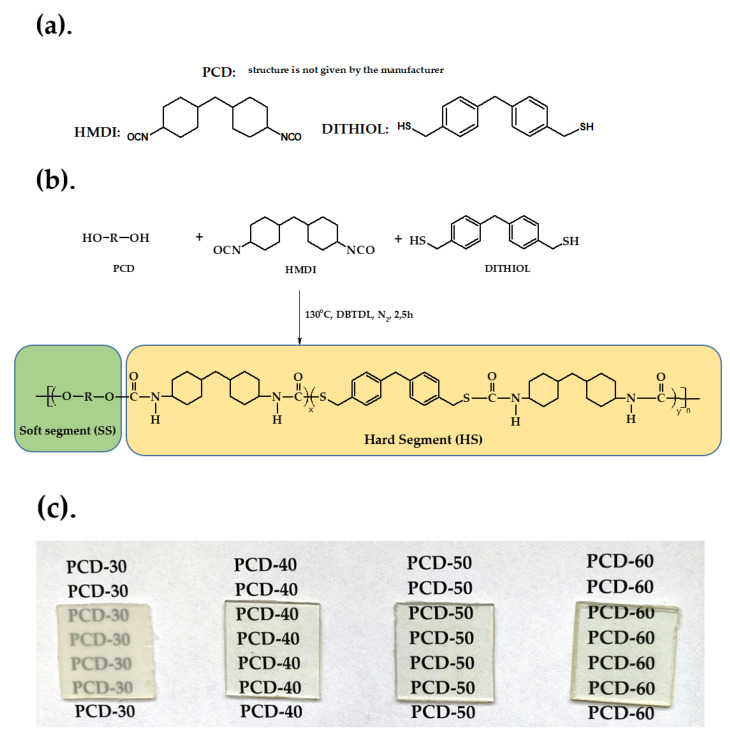
(**a**). Chemical structures of the reactants; (**b**) Schematic representation of the synthesis route of polymers; (**c**) Images of the synthesized polymers.

PTURs with hard-segment contents of 30, 40, 50 and 60 wt% were prepared by a one-step melt polymerization from dithiol, HMDI and at the NCO/(OH + SH) molar ratio of 1.07. The general procedure for the synthesis of PTURs using this method was as follows. Oligomer diol and dithiol (0.01 mol together) and HMDI (0.0107 mol) were heated with stirring under dry nitrogen to 90 °C in an oil bath. A catalytic amount of DBTDL (about 0.03 g) was added to the formed clear melt and polymerization rapidly began with vigorous stirring. The reaction temperature was gradually elevated to 130 °C and the formed rubber-like product was additionally heated at this temperature for 2 h. Schematic representation of the polymer synthesis is given in Figure 2.

Designations and composition used to synthesize the PTURs are given in Table 1. The polymers were designated as X-Y, where X is the abbreviation of soft segment and Y represents the hard-segment content.

Our use of a polycarbonate soft segment as a building block was extremely important in terms of the potential use of the newly obtained PTURs as biomedical materials. As is commonly known, depending on what the obtained material is to be used for, we can use different SS. Our goal was to obtain polyurethane materials that could be used in long-term implantation. Therefore, we chose polycarbonate among the different types of SS. Additional advantages of polyurethanes obtained with the use of PCD are their better resistance to hydrolysis than polymers obtained from polyester segments and more favorable strength properties than polymers with a polyether SS.

## 3. Results and Discussion

The polymers obtained were transparent or partially transparent (PCD-30) solids (see Figure 2c). All these polymers dissolved at room temperature in THF, TChE, chloroform, *N*,*N*-dimethylacetamide and *N*,*N*-dimethylformamide, but they were insoluble in *N*-methyl-2-pyrrolidone and dimethyl sulfoxide.

### 3.1. FTIR

**Figure 3:**
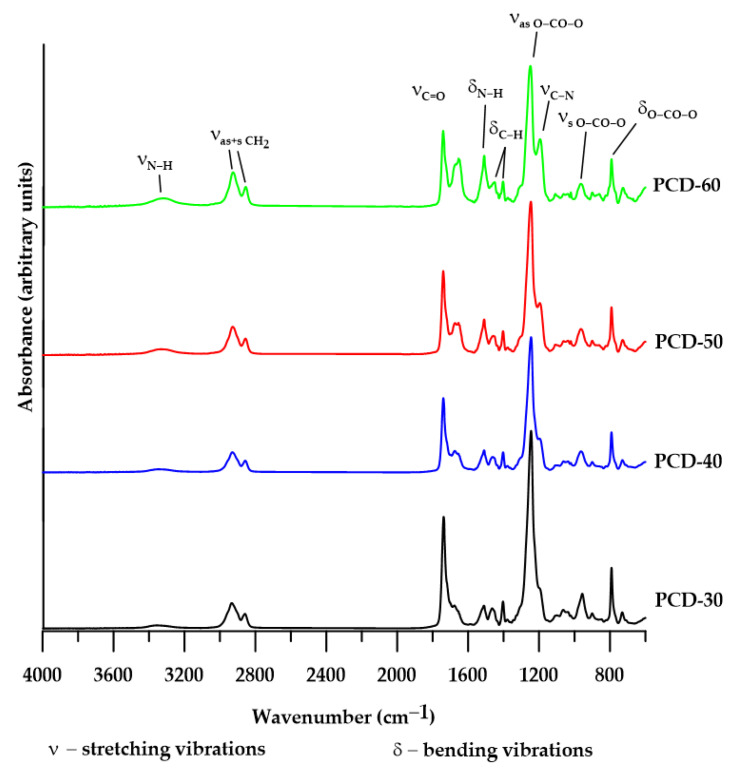
FTIR spectra of synthesized polymers.

The structures of all the polymers were verified by FTIR spectroscopy. Characteristic bands of different groups present in PTURs are visible in the FTIR spectra (see Figure 3).

On the FTIR spectra obtained for all the studied PTURs, a broad absorption band in the range 3351–3309 cm^−1^ was observed. It is typical for the N-H stretching vibrations of urethane and thiourethane groups [33,34]. For the same fragments of the above-mentioned groups, we also observe bending vibrations in the range 1511–1509 cm^−1^ [33,34]. The next clear bands visible in each spectrum are the peaks corresponding to C-H stretching vibrations of the CH_2_ group; asymmetric ones are visible in the range 2933–2926 cm^−1^, while symmetric ones in the range 2859–2854 cm^−1^. Next, we can read the bands corresponding to the C-H bending vibration of the CH_2_ group in the cyclohexane structure and in the aliphatic CH_2_; the asymmetric ones are located in the range 1465–1450 cm^−1^, while the symmetric ones are located in the range 1404–1403 cm^−1^ [33]. FTIR spectra of all the obtained polymers also indicated strong absorption in the range 1741–1738 cm^−1^ and 1676–1654 cm^−1^. The former range corresponds to C=O stretching vibrations from carbonate groups while the latter corresponds to the same vibrations in the thiourethane groups [33,34]. In the case of C=O stretching vibrations occurring in the urethane group, the band corresponding to them can only be read for the structure containing 40% of the HS—1700 cm^−1^. In the case of the other polymers, these bands are hardly visible due to the broad peak coming from the carbonate group. The next observed bands are those corresponding to the stretching vibrations of the carbonate group; for asymmetric ones—1249–1245 cm^−1^ and for symmetric ones—957–900 cm^−1^ [33]. On all spectra, we can also distinguish the bending vibration peaks of the carbonate group out-of-plane, which are in the range 792–791 cm^−1^, while in-plane, they are in the range 731–727 cm^−1^. For structures with a high content of HS (50 and 60 wt%), we can distinguish additional bands in the range 1195–1194 cm^−1^. We attribute them to C-N stretching and N-H bending vibrations. These peaks are not observed for polymer structures with lower hard-segment content [33,34].

The absence of the –NCO band at about 2260 cm^−1^ shows that all –NCO groups were converted to urethane and thiourethane groups [34]. Table 2 presents major absorption bands appearing for the FTIR spectra of the obtained PTURs.

The changes in the intensity of the individual bands are closely related to the changes in the chemical composition of different polymers.

### 3.2. Physicochemical Characterization

#### 3.2.1. Reduced Viscosities and GPC

Reduced viscosity values of the obtained polymers (given in Table 3) ranged from 0.62 to 3.63 dL/g, and in each series they decreased with increasing SS content in the sample. The obtained PTURs were materials which exhibit high molar masses ($\bar{M_{n}}$ in the range of 21,700–42,000 Da and $\bar{M_{w}}$ in the range of 33,000–62,000 Da) and relatively low dispersities (*Ð_M_* ranged from 1.43 to 1.66). The low molar mass dispersities of the obtained polymers results from the relatively long mixing time of the contents of the reaction flasks during the syntheses. This is due to the fact that at 135 °C, all polymers were in a plasticized state. This indicates the high homogeneity of the polymers obtained.

#### 3.2.2. CAs and SFE

**Figure 4:**
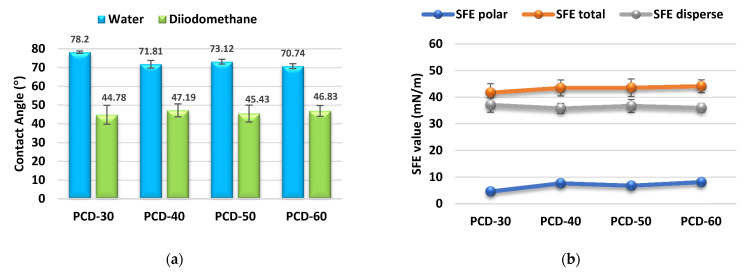
Quantifying the contact angles of (**a**) and SFE (**b**) of synthesized polymers.

The surface properties of the obtained polymers were determined on the basis of the CA and SFE values, and the obtained results are shown in Figure 4.

When using polymers as biomaterials, the chemical properties and surface properties are of key importance. Depending on the type of biomaterial used, both hydrophobic and hydrophilic properties may be advantageous. The CA and SFE values are significant in determining these properties. For polyurethanes, SFE values exceed 50 mN/m; therefore, they are classified as polar materials [63].

An important aspect for the researcher was the combination of biocompatibility with the value of critical surface energy. This problem was dealt with by Baier [71] in his research. According to the hypothesis proposed by him, the surface of the material with the so-called critical surface energy at the level of 20–30 mN/m is characterized by thrombogenicity and was defined by Baier as a hypothetical zone of biocompatibility, understood as the zone of minimal cell adhesion. On the other hand, materials with a critical surface energy above 40 mN/m promote cell adhesion. Therefore, they are good materials, especially for orthopedics, where the adhesion of cells to the implant surface and the overgrowth of the implant with tissues play a key role in the healing process.

It is commonly accepted that hydrophilic surfaces have CA values up to about 30°, and hydrophobic surfaces above 90° [63]. As it can be seen from Figure 3, all obtained PTURs were hydrophilic (CAs for water between 70.74° and 78.2°).

#### 3.2.3. Hydrolytic Resistance

**Figure 5:**
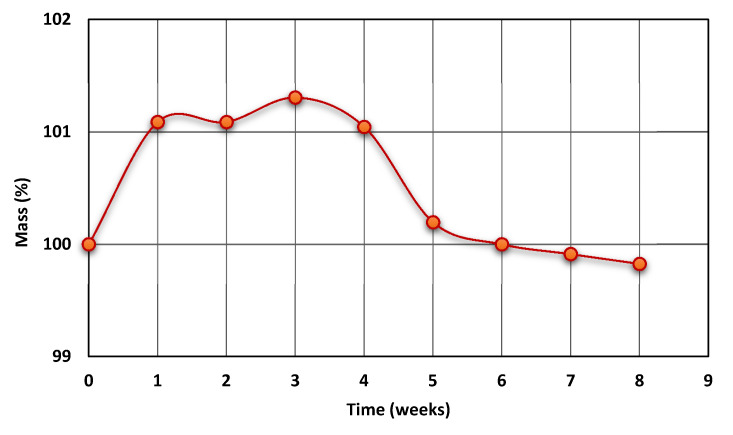
Diagram of sample mass change during Optylite^®^ liquid incubation.

The hydrolytic resistance of polyurethane material in the human body environment is extremely important in biomedical applications. To preliminarily evaluate this property, PTURs with 50% wt. content were incubated in Opylite^®^ salt solution for 8 weeks. The observed changes in the mass of the test sample are presented in Figure 5.

As can be seen in Figure 5, during the initial stage of incubation, the physiological liquid was absorbed by the polymer chain (in this stage the polymer was swollen). After appropriate saturation with the solution (and loosening of the polymer chain), the hydrolysis of carbonate bonds took place, which shows the small weight loss of the tested sample, which is visible on the curve. The conducted research shows that the PCD-50 polymer is hydrolytically stable in a multi-ion physiological fluid for about 6 weeks and its hydrolysis only takes place after this time.

It is noteworthy that after 8 weeks, the mass of the samples decreased by about 0.2%. According to the available literature, significant changes in the hydrolytic stability of polyurethanes only occur during the longer incubation period [72,73,74].

### 3.3. Thermal and Thermomechanical Properties

#### 3.3.1. DSC

**Figure 6:**
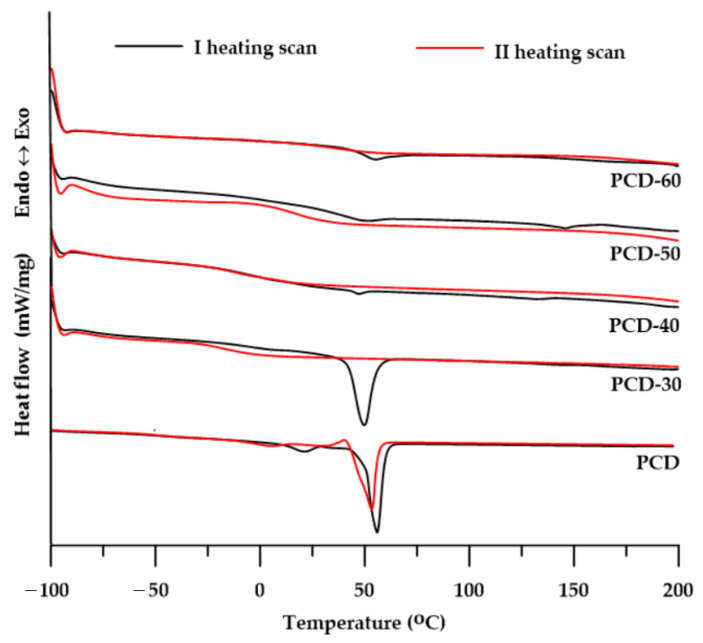
DSC curves of the synthesized polymers.

The changes in physical transformation of the obtained PTURs were determined by DSC analysis. In order to better interpret the obtained results, the compounds that constituted SS in the obtained polymers were also tested. The numerical data of the analyses are presented in Table 4, while the shapes of the DSC curves are presented in Figure 6.

The determined *T_g_* values of the obtained PTURs were in the range of −2–44 °C in the first heating cycle and −15–33 °C in the second heating cycle. These values increased with the increase in the content of the HS in the polymers, with a significant increase being observed for the polymer with 50 wt% of hard segments. Considering the differences in the *T_g_* values of polymers and pure soft segments (PCD: −40 °C), it can be concluded that with the increase in the content of the hard segment in PTURs, their microphase separation degree decreased. The *T_g_* values in the second heating are much lower than in the first heating, i.e., repeated heating of the sample lowers its glass transition temperature. Polymers with a 50 and 60 wt% of the hard segment showed *T_g_* values slightly higher than room temperature; therefore, they should be located on the border of elastomers and plastomers. More precise viscoelastic properties of the obtained materials were determined by the DMTA method and the description of the obtained results is presented in the rest of this work.

On the DSC curves of all polymers (see Figure 6) from the I heating cycle, one or two endothermic peaks with *T_m_* values in the range of 47–146 °C were observed. The peaks with the lowest *T_m_* values in the range of 47–55 °C and Δ*H* values in the range of 0.7–34.1 J/g may be responsible for the melting of the soft segment. In turn, small visible endothermic peaks with *T_m_* values ranging from 132 to 146 °C should be attributed to the melting of more or less ordered hard segments [33]. The most intense peak of SS melting is visible for the PCD-30 polymer, while for the PCD-50 and PCD-60 polymers the heat of this transformation is very low, which makes it difficult to speak of a visible peak. The DSC curves of the second heat no longer reveal any melting energy effects of SS or HS. This indicates that the intrinsic capacity of the obtained polymers is very low.

The low Δ*H* values determined from the I heating cycle and the absence of endothermic peaks in the II heating cycle curves indicate a slight tendency of the obtained PTURs to form ordered structures. The polymer with the lowest content of hard segments (PCD-30) was characterized by the highest degree of ordering, but only within the domains of soft segments.

#### 3.3.2. TGA

The thermal stability of the obtained PTURs was determined by means of thermogravimetric analysis carried out under an inert gas atmosphere.

As can be seen from the data in Table 5, the obtained polymers had higher thermal resistance compared to the regular polymer whose TG analysis was presented in a previous work [30]. Analyzing the effect of changing the HS content in PTURs, it can be seen that its increase in the polymer caused a decrease in *T*_5_, *T*_10_ and *T*_50_.

**Figure 7:**
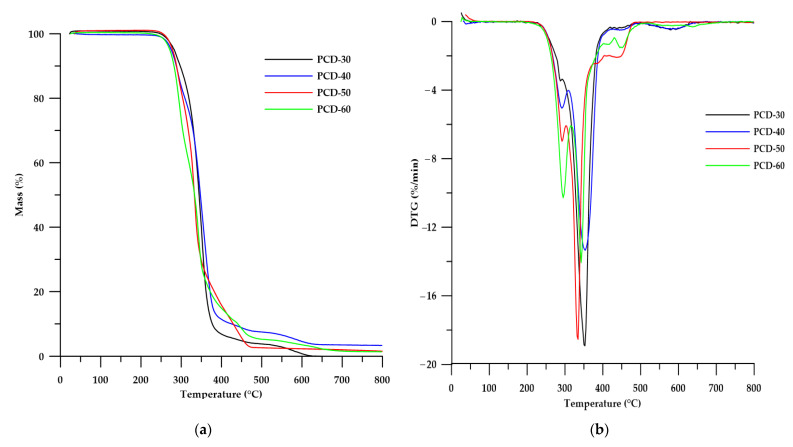
TG (**a**) and DTG (**b**) curves of the PTURs.

Based on previous studies [33], and by analyzing the data in Table 4, it can be said that the decomposition process of the obtained PTURs took place in several stages. The DTG curves shown in Figure 7 revealed relatively intense peaks at the maximum in the range of 286–295 °C, which were associated with the decomposition of thiourethane bonds. The intensity of this peaks increased with increasing HS content in the polymer. The further peaks with maxima at 333–342 °C can be attributed to the decomposition of urethane bonds. A third degradation step for polymers with an HS content below 60 wt% (peaks at about 362–367 °C) is related to the degradation of the polycarbonate soft segment. The last decomposition step for all polymers (peaks at about 429–451 °C) can be attributed to the decomposition of the aromatic structure fragments of the obtained PTURs [33,34]. The small peaks seen at temperatures around 600 °C may be related to the decomposition of the solid products formed in the earlier stages.

#### 3.3.3. DMTA

**Figure 8:**
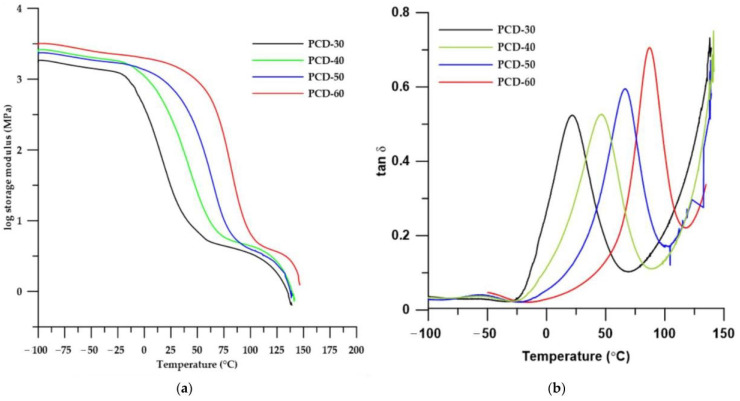
Temperature dependence of storage modulus (**a**) and tanδ (**b**) of synthesized PTURs.

To investigate the influence of the soft segments on the viscoelastic properties of PTURs, dynamic mechanical thermal analysis was performed. Changes in storage modulus (*E*′) and mechanical loss factor (tanδ) with temperature are shown in Figure 8, while DMTA data are summarized in Table 6.

As can be seen from the data in Table 6, with the increase in the content of HS in the polymer, almost all parameters determined by the DMTA method increased. The exception was the damping value (tan*δ*_max_) and the FWHM parameter (full width at half maximum), which is a degree of sample homogeneity.

On the basis of the results obtained, it can be stated that as the content of HS in the polymers increased, their glass transition temperatures increased, which is in agreement with the results of the DSC analysis. Increasing the value of the storage modulus in the series of polymers causes materials to become harder and stiffer. These conclusions were confirmed by the results of the strength test results described later in this paper. On the other hand, the decrease in the value of the FWHM parameter in the series confirms the results of the DSC, which found that as the HS content increased, hard and soft segments decreased, causing the polymer to have a more homogeneous structure.

Considering the curves of the dependence of the storage modulus on the temperature (Figure 8a), it can be concluded that, among all the materials, the polymers PCD-30 and PCD-40 exhibit the most favorable viscoelastic properties. This is due to the fact that these polymers possess rubber-like properties (i.e., they are in the rubber-elastic state) in the widest temperature range. The occurrence of this state over a wide temperature range is characteristic of elastomeric materials. The other polymers are characterized by the fact that the melting area begins almost immediately after the transition from the glassy state, in which the polymers transform into a melted amorphous state. In this state, polymers can be easily processed using conventional processing methods, such as injection molding, extrusion or 3D printing.

The tan delta vs. temperature curves show two peaks corresponding to two types of relaxations. The first one (with maxima between −60 and −50 °C) is connected to the main chain movements of the soft segment and local movements of thiourethane and urethane polar groups. The second type of relaxation (called primary relaxation) with maxima between 21 and 87 °C is related to the glass transition of the soft segment in the polymer. The intensity of the principal relaxation peak determines the material’s ability to damp (absorb) vibrations; the smaller its value, the greater the material’s damping capacity [32].

### 3.4. Mechanical Properties

The hardness, tensile strength, elongation at break, modulus of elasticity and lap shear strength are listed in Table 7.

Shore hardness of the obtained PTURs was determined on two scales: A and D. On the A scale it was in the range of 71.75–96.25 ShA, while on the D scale it was in the range of 25.00–66.00 ShD. It depends on the composition of the polymer. In general, the higher the content of the hard segment, the higher the hardness values of the polymers defined in both scales. The exception is the PCD-60 polymer, the hardness of which on the A scale is lower than that of the PCD-50 polymer, while at such high values the results can be misleading due to the near maximum range of the measurement scale.

All of the obtained polymers exhibit relatively high tensile strengths ranging from 34.43 to 51.11 MPa, which generally increased with increasing hard segment content. The elongation at break is in the range of 75–350%. The value of this property decreased as the hard segment content increased. For the modulus of elasticity, the values are in the range of 1.82–289.88 MPa. The modulus values are in agreement with the determined *T_g_* values. The PCD-30 and PCD-40 polymers that had *T_g_* below room temperature also have moduli typical of elastomers. The other two polymers that had *T_g_* values near room temperature also have significantly higher modulus values. The polymer with the highest content of the hard segment and the highest *T_g_* value (PCD-60) is also characterized by the highest value of the modulus of elasticity and the highest hardness on the D scale.

The analysis of the influence of the hard segment content on the adhesion properties to copper showed that the more content there is, the greater the strength of the adhesive joint. This dependence confirms that with an increase in the amount of extender, and thus with an increase in the amount of sulfur atoms in the polymer, the affinity (adhesion) of such material to copper increases. This is in accordance with previous studies, and the values achieved for the obtained materials were higher than those previously described in the literature [33,34,38,49,58].

### 3.5. Optical Properties

#### 3.5.1. Refractive Index and Transparency

The obtained polymers had refractive index values ranging from 1.5135 to 1.5615 (see Table 8), and these values increased with increasing HS content in the polymer. This relationship is in agreement with previous results, which confirm that the refractive index value depends on the presence and amount of sulfur atoms in the polymer; the more sulfur atoms there are, the higher the refractive index of the polymer.

In contrast to the refractive indexes, the transparency of the obtained polymers decreased with increasing HS content in the polymer. The exception was the polymer PCD-30, which was nontransparent (see Figure 2c). The polymer with the highest transparency was PCD-40, with little difference compared to the transparency of PCD-50. The PCD-30 polymer had the lowest transparency, which was due to the relatively high degree of ordering within the soft segment domains. To further characterize the color dependence of the HS content in the polymer, a color test according to ASTM E308 was performed.

#### 3.5.2. Color

**Figure 9:**
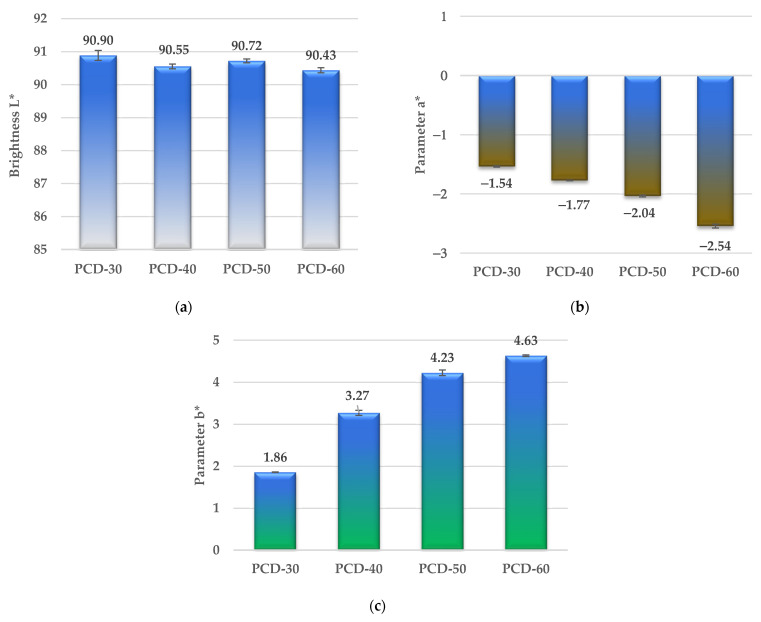
Brightness L* (**a**), parameter a* (**b**) and parameter b* (**c**) of synthesized PTURs.

The results of the color tests of the obtained polymers are shown in Figure 9.

As can be seen from Figure 9a, the brightness L* of the polymers in the series generally decreases (except for the PCD-50 polymer), which indicates that the proportion of white color in the series decreases. On the other hand, the increase in parameter b* in the series causes that the proportion of yellow color to increase, which can be seen by observing the appearance of the samples in Figure 2c. The increase in parameter b* in the series can be related to the increase in the number of sulfur atoms in the polymer, i.e., the more sulfur there is, the more yellow the polymer. A similar relationship was observed for parameter a*, as the increase in parameter a* causes a shift in the color of the obtained polymer towards the green color.

## 4. Conclusions

The use of a one-step melt polyaddition method, carried out with a slight excess of isocyanate groups to hydroxyl and thiol groups (NCO/(OH + SH) = 1.07), enabled the preparation of high-molecular PTURs. The obtained polymers were colorless opaque rubber-like solids with relatively high transparency. PCD-40 and PCD-50 polymers exhibited similar and best transparency. The synthesized PTURs were soluble at room temperature in DMF, TChE and THF. All polymers of NMP and DMSO showed high resistance.

The *η*_red_ values determined were in the range of 0.62–3.63 dL/g. It was observed that as the hard segment content increased, the viscosities of the obtained PTURs decreased. The values determined $\bar{M_{n}}$ and $\bar{M_{w}}$ for THF-soluble polymers were in the range of 21,700–42,000 Da and 33,000–62,000 Da, respectively. The dispersity of molar masses contained in the range 1.46–1.66 was quite low for the polymers obtained by the one-step melt method. This indicates that their structures were highly homogeneous. After examining the refractive index, it was found that as the content of the hard segment in the polymer increased, the value of this index increased.

The TGA investigations allow us to conclude that the obtained PTURs were characterized by relatively good thermal stability. From the values obtained, it can be seen that the mass loss temperatures decreased with the increase in the hard segment content. DSC analysis showed that the obtained PTURs exhibit a partially ordered structure. The determined *T_g_* values were in the range of −2–33 °C in the I heating cycle and −15–44 °C in the II heating cycle. These values increased with increasing hard segment content in the polymers. From the obtained *T_g_* values, it was concluded that the obtained PTURs were elastomers or plastomers. DMTA analysis confirmed these observations.

Upon examination of the mechanical properties, it was found that all the obtained polymers exhibited relatively high tensile strengths (34.43–51.11 MPa), which increased with the increase in the hard segment content. The same relationship was exhibited by the shore hardness values. On the other hand, the elongation at break decreased with the increase in hard segment content. From the PTUR adhesion tests, it is evident that as the hard segment content in the polymer increases (and thus the sulfur content increases), the adhesion strength increases.

## Figures and Tables

**Table 1:** Designations of the PTURs.

PTUR	Amount of Dithiol (mol %)	Amount of Soft Segment (mol %)	^1^ Hard-Segment Content (wt%)
PCD-30	53	47	29.87
PCD-40	67	33	39.83
PCD-50	77	23	50.15
PCD-60	85	15	59.88

^1^ The hard-segment content (wt%) is calculated by using the expression $\frac{W_{dithiol}+W_{HMDI}}{W_{dithiol}+W_{HMDI}+W_{SS}}$, where *W_dithiol_*, *W_HMDI_* and *W_SS_* are the mass of dithiol, HMDI and soft segment, respectively.

**Table 2:** Basic absorption bands appearing for the FTIR spectra of the obtained PTURs.

	Types of Vibrations [cm^−1^]													
PTUR	ν_N–H_	ν_C–H_ CH_2_		ν_C=O_ Carbonyl	ν_C=O_ Thiour.		δ_N–H_	δ_C–H_ 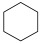 and CH_2_		ν _O–CO–O_ Carbonyl		ν_C–N_ + δ_N–H_	δ _O–CO–O_ Carbonyl	
		ν^as^	ν^sym^		H-Bonded	Free		ν^as^	ν^sym^	ν^as^	ν^sym^		Out of Plane	in pl.
PCD-30	3351	2933	2859	1738	-	1676	1511	1465	1404	1245	957	-	791	731
PCD-40	3351	2930	2857	1740	-	1676	1510	1458	1403	1245	901	-	792	730
PCD-50	3338	2928	2856	1740	1654	1672	1510	1458	1403	1246	900	1195	792	729
PCD-60	3309	2926	2854	1741	1654	-	1509	1450	1404	1249	900	1194	791	727

**Table 3:** *η*_red_ values and GPC data of the PTURs.

PTUR	*η*_red_ (dL/g)	$\bar{M_{n}}\;(\mathrm{Da})$	$\bar{M_{w}}\;(\mathrm{Da})$	*Ð_M_*
PCD-30	3.63	41,900	60,100	1.43
PCD-40	1.90	32,000	53,200	1.66
PCD-50	1.54	42,000	62,000	1.48
PCD-60	0.62	21,700	33,000	1.52

**Table 4:** DSC data of the synthesized PTURs and SS.

PTUR	*T*_g_ [°C]		*T*_m_ [°C]		Δ*H* [J/g]	
	I ^a^	II ^b^	I ^a^	II ^b^	I ^a^	II ^b^
PCD-30	−2	−15	50	-	34.1	-
PCD-40	1	−12	47; 132	-	0.7; 0.6	-
PCD-50	32	20	52; 146	-	1.9; 2.2	-
PCD-60	44	33	55	-	2.6	-
PCD	−40	−50	21; 56	5.6; 40.1; 53.3	6.6; 64.8	3.6; 3.3; 53.0

^a, b^ first and second heating scan, respectively.

**Table 5:** TG data of the synthesized PTURs.

PTUR	*T*_5_ ^1^ (°C)	*T*_10_ ^2^ (°C)	*T*_50_ ^3^ (°C)	*T_max_*^4^ (°C)
PCD-30	281	295	343	286; 333; 360; 429
PCD-40	279	289	341	292; 339; 362; 444
PCD-50	278	288	337	292; 334; 367; 446
PCD-60	275	285	333	295; 342; 451

^1, 2, 3^ The temperatures of 5%, 10% and 50% mass loss, respectively; ^4^ The temperatures of maximum rate of mass loss.

**Table 6:** DMTA results of the PTURs.

PTUR	*E*′_onset_ (°C)	*E*′*_20_* (MPa)	*E*″_max_ (°C)	T _tan*δ*max_ (°C)	tan*δ*_max_	FWHM (°C)
PCD-30	−6.69	52	−5.81	21.07	0.459	48.45
PCD-40	10.55	418	5.81	46.19	0.448	40.23
PCD-50	36.98	910	27.42	66.37	0.461	31.43
PCD-60	61.41	1704	50.95	86.87	0.535	24.48

**Table 7:** Hardness and mechanical properties of PTURs.

PTUR	Hardness (Sh)		Tensile Strength (MPa)	Elongation at Break (%)	Modulus of Elasticity (MPa)	Lap Shear Strength (MPa)
	**A**	**D**				
PCD-30	71.75 ± 1.50	25.00 ± 0.82	34.43 ± 0.23	350 ± 0	1.82 ± 0.13	4.32 ± 0.33
PCD-40	84.75 ± 0.50	34.25 ± 2.99	45.76 ± 2.71	275 ± 0	4.35 ± 0.56	14.70 ± 0.51
PCD-50	96.25 ± 0.50	53.00 ± 1.87	46.54 ± 4.06	223 ± 2.89	69.51 ± 2.19	15.93 ± 0.64
PCD-60	91.50 ± 1.15	66.00 ± 1.41	51.11 ± 2.37	75 ± 0	289.88 ± 2.88	18.05 ± 0.44

**Table 8:** Refractive index and transmittance of PTURs.

PTUR	Refractive Index	Transmittance (%)	
		*T*_500_ ^1^	*T*_800_ ^2^
PCD-30	- ^3^	63.41 ± 0.012	71.78 ± 0.009
PCD-40	1.5135 ± 0.002	76.52 ± 0.008	82.91 ± 0.014
PCD-50	1.5355 ± 0.003	76.05 ± 0.007	82.89 ± 0.011
PCD-60	1.5615 ± 0.003	72.67 ± 0.010	80.15 ± 0.007

^1, 2^ transmittance at 500 and 800 nm, respectively. ^3^ opaque.

## Data Availability

The data presented in this study are available on request from the corresponding author.
